# National Dental Association

**Published:** 1899-11

**Authors:** 


### Reports of Society Meetings.

### NATIONAL DENTAL ASSOCIATION.

(Continued from page 666.)

*Second Day.—Morning Session.*

SECTION III., Operative Dentistry, was called, and Dr. J. T. Crawford, chairman, made a partial report, offering a paper by Dr. N. S. Jenkins, Dresden, Germany, entitled "Porcelain Enamel Inlays."

Dr. Jenkins then read his paper, of which a brief abstract follows:

The process and material presented is the result of seven years of study and experiment, progressing through its various stages by at once trying in the mouth the product of the laboratory, scientific experiment and practical application going hand in hand. The successful use of this finally completed method for over two years by a considerable number of the foremost dentists of the world justifies the announcement that the problem of making, with mathematical accuracy and scientific certainty, absolutely perfect fillings in diseased teeth has been completely solved. As to what constitutes a perfect filling: it must fill the cavity so exactly as to exclude moisture; it must be of a substance which will not disintegrate nor change its original form either through chemical action or mechanical force; it must have a surface so smooth that it can be easily kept clean; it must be a poor conductor of caloric; it must retain the color and shape of the teeth; it must be applicable to the most desperate cases and susceptible of being used without too great strain upon timid children and delicate patients, as well as ordinary patients; and its working must not make too great draughts upon the strength and nerves of the operator. Last of all, it must be possible for any good dentist to use it with the certainty of obtaining infallible results. All these qualities are possessed by the material which I have called porcelain enamel. A substance which can be melted in a gold-foil matrix is necessary, in order to permit its use by any competent dentist in cavities in any part of the mouth, as an ordinary and regular proceeding in daily practice. Only the most exceptionally gifted and patient man can obtain such results with platinum, owing to its intractability. A perfect impression is the indispensable foundation of a perfect inlay. Gold-foil No. 30 seems best adapted to the great majority of cavities. To keep the gold-foil impression exact during fusing a paste of powdered asbestos and water is found best adapted to hold the impression in place in the melting-pan, evaporating the moisture gently in drying out. The heat of gas is insufficient for fusing. The porcelain enamel has sufficient strength to withstand the force of mastication, and a surface which resists all chemical action except that of hydrofluoric acid. It fuses at a temperature of 800° to 900° C., the melting-point of gold being 1075° C. This difference is sufficient to prevent melting the gold matrix unless through great carelessness; it also prevents the gold-foil from adhering to the porcelain enamel, from which it is easily stripped. The fit of the inlay is so exact that only a slight film of cement is necessary for retaining the filling, but it is important to groove the inlay with a small diamond disk, and to form small undercuts in the cavity before setting the inlay.

In using phosphate cement in setting inlays it is well to saturate the cavity with carbolic acid, drying it out after a few minutes, rendering the pulp less sensitive to the irritating action of the phosphoric acid. A cement with very fine powder, and which does not suddenly crystalize, is to be preferred. The powder composing the inlay should be mixed with absolute alcohol, which evaporates with less disturbance of the particles than water, and because it carries no deleterious substance in solution. In the selection of color it is well, for small inlays, to choose a color slightly darker than the tooth except for proximal cavities, where a lighter color is often indicated. Selecting the color after the tooth has been dried under the rubber dam is always misleading; the color must correspond with that of the tooth in its natural moist condition.

In the beginning the operator must be prepared to give this work more time than for his accustomed method of gold filling, but the work is less exhausting, more remunerative, and far more satisfactory, and is more merciful to the patient. The porcelain enamel adheres fairly well to platinum, and where roots are to be banded for crowns the visible portion of the band can be covered with the enamel to great advantage.

DISCUSSION.

*Dr. John I. Hart* said that he was impressed with the feeling that this Association owes a debt of gratitude to Dr. Jenkins for this exposition of his method. The interest manifested in this high class of work is a refutation of the idea that dental science is drifting towards commercialism; that it is lacking in æsthetic refinement. Individually Dr. Hart said he preferred a higher fusing body in a platinum matrix, which if properly annealed does not offer the difficulties portrayed in the paper. By the use of platinum as a matrix it is possible to try it in the cavity, and if the contour is not sufficient more body can be added, which is impracticable by Dr. Jenkins's method. In order to carry the platinum into deep cavities, Dr. Hart's method is to take an impression of the tooth and cavity in modelling compound, pouring oxyphosphate of zinc into the impression, thus obtaining a replica of the tooth and cavity which will stand the burnishing of the platinum with the cavity and over the margins. By packing body into this until it is two-thirds full. When cool it can be carried to the cavity in the tooth and pressed home, thus securing a perfect adaptation. This is better than to form the matrix in the cavity of the tooth. When the cavity is extensive, hydrofluoric acid flowed over the inner surface of the inlay will roughen it and thus enable a better hold for the cement.

*Dr. Darby* said there was no subject he was more interested in than that of porcelain inlays. He had had ample opportunity of observing Dr. Jenkins's artistic methods, and to say that he was charmed would not half express his opinion of the beauty, accuracy of fit, and perfection of color which so perfectly fulfils the needs of the case. At a distance of twelve inches it is not possible to detect which tooth has an inlay, or to distinguish the porcelain from the natural enamel. Of course it requires experience to make it perfect, but by this method a perfect inlay can be made, and it is one of the greatest of recent improvements in dental practice. It is not a method for the careless man; care is required at every step, but any one who is competent to practise dentistry can, with painstaking efforts, obtain results which will be eminently satisfactory to both himself and his patients.

*Dr. C. N. Johnson* said the profession was greatly indebted to Dr. Jenkins for his well-cut, clearly defined, easily understood description of his methods, but a word of caution is necessary, for it should not be supposed that this method is one that can be readily picked up by the average practitioners. There is nothing in operative dentistry that requires such absolute accuracy in the minutest details as this work. It is advisable that a man should know what he is undertaking, and not go into it unadvisedly. The requirements are severe in every detail. The ordinary operator will have his gold filling completed in less time than is necessary to prepare the cavity and make the matrix for a porcelain inlay of this character. When a gold filling is properly made you know it will remain there; there will be no leak at the joint, for there is no cement to dissolve out; it is in perfect contact at every point. So while we deplore the show of gold in the mouth, a gold filling is nevertheless preferable for many reasons.

*Dr. Joseph Head* said that Dr. Johnson's remarks reminded him of the French woman who regretted that she could not have her teeth filled in New York, because the New York dentists used nothing but gold. The reply was made that gold was the only permanent filling, but the lady said she preferred a beautiful white filling to one that was merely permanent.

As to permanency, we know that inlays have lasted and do last; even the old glass fillings, which turned dark by the action of the saliva, have lasted eight and ten years, and seem likely to last eight or ten years longer. While it is true that some dentists succeed better than others, any dentist who is capable of putting in a good gold filling is capable of putting in an inlay. The average life of a gold filling is not over five years, and why should we ask for more from porcelain inlays? Of course, they cannot be put in with the ease with which you would pack in gutta-percha. The man who undertakes it will have many disappointments before he reaches success, but let him look back to his first gold fillings and compare them with his present work, and he will not expect his first inlay to be satisfactory or perfect.

The question is not, however, whether a good operator can do this work, but it must be borne in mind that the Jenkins porcelain is only two years old in its present perfected form, or three at the most; that it is a low-grade fusing body. I hold that at present the method and the material are on trial. It is comparatively new and untried. We are not yet prepared to pass judgment upon it. All porcelain when hot contracts, and there is the risk of distorting the matrix; but with a platinum matrix it can be partially filled, and when it is replaced in the cavity the edges can be burnished to the cavity walls again and more porcelain added, making the edges almost perfect. This possibility of a second burnishing seems very desirable. The question of choice between a low- or a high-fusing body resolves itself into that of whether a better adaptation can be secured by a platinum or a gold matrix. If platinum is badly treated and not properly annealed, it is a harsh material, but when properly annealed it is about as soft as lead, so that there is no difficulty in obtaining a perfect matrix with it. Whatever method be adopted, mechanical skill and due care will give good results; but without this, good results cannot be expected from any system. I believe, however, that with equal skill and care in the long run a high-fusing body in a platinum matrix will give the greatest satisfaction.

*Dr. George Evans* said that he had recently been so fortunate as to secure Dr. Jenkins's set of enamels and inlay materials. He had not yet had the opportunity of testing the inlays, but had made some experiments with the process in enamelling gold crowns, producing æsthetic results which he would exhibit at a clinic. Dr. Jenkins's porcelain offers great advantages in enamelling the labial surfaces of all-gold crowns, the gold crown with properly strengthened cusps offering great advantages in perfect adaptation, and when enamelled in the exact shade required, as can be done with the Jenkins porcelain enamel, the result compares favorably with the English tooth.

He had investigated the question of high- or low-fusing bodies from an absolutely disinterested stand-point, although prejudiced against the low-fusing body; but after seeing the wonderful margins, the close joint, the strength and the simplicity of the process, he is now convinced that the Jenkins material is fully equal in these respects to any high-fusing body. Personally he cannot speak as to its durability, but he is willing to take the word of such men as Dr. Jenkins and Dr. Darby.

*Dr. H. J. McKellops* said that, as is well known, he is emphatically *a gold worker.* For the corner of a front tooth nothing can equal platinum gold; nothing can touch it; you cannot wear it out; with nothing else can you accomplish such grand results, and he is astonished in travelling the world over to find that it is not more generally used. Porcelain inlays can probably be made with greater ease and comfort to the patient, but when you want durability, platinum-gold is the thing. It gives absolute satisfaction in every way.

*Dr. Ottolengui.*—Discussion is a failure when it does not reach a finality, when only the same old statements are reiterated on both sides. Such discussion convinces no one. It still remains a matter of personal opinion whether we shall use a low-fusing body in a gold matrix or a high-fusing body in a platinum matrix. We are told by the advocates of the latter that it can be returned to the cavity and burnished a second time, thus overcoming shrinkage. One side says this cannot be done with a gold matrix; the other side says that it can. Platinum requires reburnishing because it does not make a perfect matrix. In any cavity where a gold matrix can be adopted perfect results can be attained. An amalgam filling which occupied the anterior proximal and occlusal surface was removed from a molar tooth. This was replaced with a Jenkins inlay, made in a gold matrix. When it was inserted no fault could be found with the edges. He does not believe that it could have been done with a platinum matrix. It is proposed that duplicate cavities be prepared and filled by the different methods respectively, and have the question definitely settled once for all.

*Dr. Jenkins,* in closing the discussion, said that the special claim he made for his system was its *simplicity,* without in any way questioning the fact that equally good results could be obtained by other methods, but at the expense of more time and greater labor; but why spend the time in laborious methods when it can be done in a more simple, easy, and rapid manner? In regard to Dr. McKellops's remark, there is no doubt but that he attains magnificent results with platinum-gold, but there are but few who have his skill in handling platinum-gold. Restorations in porcelain enamel are the nearest approach to nature possible to dental art.

